# Health-Promoting Properties of Borage Seed Oil Fractionated by Supercritical Carbon Dioxide Extraction

**DOI:** 10.3390/foods10102471

**Published:** 2021-10-15

**Authors:** Lourdes Casas-Cardoso, Casimiro Mantell, Sara Obregón, Cristina Cejudo-Bastante, Ángeles Alonso-Moraga, Enrique J. Martínez de la Ossa, Antonio de Haro-Bailón

**Affiliations:** 1 Department of Chemical Engineering and Food Technology, Faculty of Science, International Campus of Excellence in Agrifood, ceiA3, University of Cadiz, Box 40, 11510 Puerto Real, Spain; casimiro.mantell@uca.es (C.M.); cristina.cejudo@uca.es (C.C.-B.); enrique.martinezdelaossa@uca.es (E.J.M.d.l.O.); 2 Plant Breeding Department, Institute of Sustainable Agriculture, Spanish National Research Council—CSIC, 14080 Córdoba, Spain; saraobregon@ias.csic.es (S.O.); adeharobailon@ias.csic.es (A.d.H.-B.); 3 Genetic Department, Gregor Mendel Building, Faculty of Science, University of Córdoba, Campus Rabanales, 14014 Córdoba, Spain; ge1almoa@uco.es

**Keywords:** borage oil, supercritical carbon dioxide, precipitation in two cyclone separators, countercurrent extraction column, antioxidant capacity, cytotoxic activity, HL 60 leukaemia cells

## Abstract

Borage (*Borago officinalis* L.) seed oil is an important source of γ–linolenic acid, which is normally used as a treatment against different pathologies. Since the fractionation of this interesting seed oil has many environmental, economic and biological benefits, two borage fractionation techniques after extraction with CO_2_ under supercritical conditions have been studied: precipitation in two cyclone separators and countercurrent extraction column. Both techniques have successfully collected free fatty acids in one fraction: (i) two separators set up in series obtained the highest concentration of free fatty acids in separator 2 at 90 bar/40 °C; (ii) when countercurrent extraction column was used, the acidity index of the raffinate stream was independent from the operating conditions (2.6 ± 0.5%). Furthermore, the composition of the fatty acids, as well as their antioxidant and cytotoxic activities, were determined. The profile of the fatty acids obtained by either of these two methods remained unaltered, so that the crude oil exhibited improved antioxidant and cytotoxic properties. All the extracts obtained in the two cyclone separators at the same pressure/temperature conditions displayed high tumouricidal activity against HL 60 promyelocytic leukaemia cells, even if the extracts at 50% concentration from separator 2 presented a lower inhibitory activity (IC_50_). The extracts from separator 2 at 90 bar/40 °C exhibited the highest anti-proliferative activity at low doses (IC_50_ of 0.3 μL/mL for the trypan blue exclusion test). To reach the lethal dose—IC_50_—with the product obtained through countercurrent column fractionation, a concentration of 2 μL/mL of crude borage oil raffinate was required.

## 1. Introduction

Seed oils are a complex mixture of lipids, and their main component are triglycerides. However, oils also contain mono and diglycerides, free fatty acids, phospholipids, glycolipids, sterols, and other components, including tocopherols, carotenoids and phenolic compounds [1]. Oils and their lipid components are widely used in the food industry but also in the cosmetic and pharmaceutical sectors. Functional food and nutraceutical markets have been recently targeting bioactive lipid components, such as ω-3 and ω-6 fatty acids, phytosterols, tocopherols and tocotrineols because of their health benefits. Therefore, more investigations on high specificity and selectivity are required to obtain fractions that are enriched with these bioactive compounds.

Borage (*Borago officinalis* L.) is an annual plant originally from the Mediterranean region that has been used from ancient times for culinary and medicinal purposes [2]. Considerable interest in borage has been recently renewed, since its seeds seem to be the richest known source of gamma linolenic (all *cis*-6,9,12 octadecatrienoic) acid (GLA), representing between 16 and 28% of the oil content in the seeds, which makes between 27 and 37% of the whole seed. Diets are supplemented with vegetable oils containing GLA, in those cases where health problems are associated to GLA deficiency [3,4,5,6].

GLA is used for the prevention and treatment of degenerative pathologies such as diabetes [7], osteoporosis [8] or cancer [9,10]. It has also been used to treat arthritis, certain skin disorders (i.e., atopic eczema), reproductive disorders, including breast pain and premenstrual syndrome, cardiovascular diseases and neurological problems related to diabetes [11,12].

Borage seed oil has been promoted as an effective treatment for different pathologies, such as rheumatoid arthritis, diabetic neuropathy, atopic dermatitis, premenstrual syndrome, and menopause-related symptoms [13,14]. The protective effect of commercial borage seed oil and GLA on DNA has also been demonstrated based on their antimutagenic effect and also on its in vitro cytotoxic activity towards promyelocytic HL 60 cells [15]. It has recently been confirmed that borage seed oil treated with lipase can be used as a natural skin whitening cosmeceutical product [16].

Due to current environmental concerns, environmentally friendly, both extraction and fractionation processes, with the lowest possible use of auxiliary chemicals as possible are to be applied. Supercritical carbon dioxide (SC-CO_2_) has been extensively used since the 1980s for specialty oil recovery as an alternative to the organic solvents (hexane) that are used in oil conventional processing. This is especially important for functional foods and the nutraceutical market since consumers are demanding “natural” products that are free from organic solvents. Numerous studies have, therefore, evaluated the impact of SC-CO_2_ conditions on the yield and composition of the oils extracted from various sources [17,18,19,20,21].

Supercritical fluid extraction from borage seed oil has been reported in various studies. Thus, Molero and Martinez de la Ossa [22] evaluated the yield and composition of borage oil obtained from its seeds using two extractions methods: SC-CO_2_ at 300 bar and 40 °C and conventional extraction process using hexane as solvent. The extraction yields were similar (26.0 wt.%); however, the quality of the oil extracted by SC-CO_2_ was superior. The composition of the borage seed oil extracted by supercritical extraction is particularly rich in unsaturated fatty acids, especially γ-linolenic acid (21.7%). For this reason, borage seed oil extracted using SC-CO_2_ could be an improvement compared to other conventional processes, since it considerably simplifies the oil refinement stages and does not require a solvent distillation stage at all. Both practices being the costliest processing stages in terms of energy consumption [22].

Soto et al. described the extraction of borage seed oil by SC-CO_2_ and the further extraction of antioxidants from the SC-CO_2_-defatted borage meal using different solvents (water, methanol, ethanol and ethyl acetate). The optimal conditions for oil extraction were established at 30 and 50 °C under 200 bar for 2.5 hours and with a continuous 1.5 L/h CO_2_ flow. On the other hand, the most potent antioxidant extracts were obtained from the SC-CO_2_-defatted borage meal at 30 °C under 200 and 150 bar pressure when water or methanol were respectively used [23].

As previously mentioned, one of the advantages of SC-CO_2_ extraction is that it does not require any refining steps, which is necessary when the process is carried out with hexane, since many of the components that are removed during conventional refining, such as phospholipids and chlorophyll, are not extracted by SC-CO_2_. However, when using SC-CO_2_ as the solvent, a fractionation procedure following the extraction is a convenient alternative if fractions enriched with specific compounds are to be obtained. A variety of techniques have been used over the last decade for the processing of oils for fractionation purposes [24,25].

Thus, the fractionation of lipid mixtures can be achieved through different approaches. For example, different authors investigated the fractionation using several separators in series and under a decreasing pressure sequence. Once the extracts are dissolved in the supercritical medium, the separator conditions would be adjusted to decrease SC-CO_2_ density, so that different compounds are collected into each separator. This procedure allows the use of a single extraction vessel to obtain several fractions with different properties. This technique has been successfully applied to separate waxes from essential oils [26], to concentrate the active ingredients in the extracts [27], and to fractionate sunflower leaf extracts [28,29,30].

Another approach to fractionation would be a fractionation column can be used for the separation of the liquid mixture feeds [31,32]. Some applications use SC-CO_2_ for the production of phytosterol esters from soybean oil deodorizer distillates [33] and canola oil deodorizer distillate [34], fatty acids from palm oil deodorizer distillates [35], and squalene from palm fatty acid distillates [36]. In addition, the countercurrent extraction and fractionation of a range of fish crude oils using SC-CO_2_ and carbon dioxide + ethanol mixtures have been studied [37]. Furthermore, the countercurrent fractionation method with SC-CO_2_ has been recently applied to separate limonene and triglycerides from the oil mixture in a waste model of citrus fruit [38].

To the best of our knowledge, no reports involving the fractionation of borage oil with SC-CO_2_ and their separation by precipitation into different cyclone separators or by countercurrent extraction column are available at present. Our hypothesis is that these techniques can be used to improve the selectivity and specificity of the extraction process. Therefore, the main objective of this work is to evaluate the health-promoting properties of the borage seed oils that have been obtained by applying either of these two supercritical fluid fractionation techniques. The chemopreventive activity of the different extracts obtained through borage seed oil fractionation were evaluated by conducting cytotoxicity assays against HL 60 leukaemia cells.

## 2. Materials and Methods

### 2.1. Raw Material and Chemicals

The genotype of borage Bo IAS-01 that has been analysed in this study is part of the Boraginaceae germplasm collection at the Institute of Sustainable Agriculture (IAS-CSIC, Cordoba, Spain). This line was selected because of its good agronomic performance in previous studies carried out under the typical rainfed conditions of Mediterranean ecosystems [5,39]. The plants were cultivated in an experimental orchard at the IAS (37.8° N 4.8° W) in a sandy-loam soil, (classified as a Typic Xerofluvent). After ripening, the seeds were harvested, washed, and shipped over to UCA (Cadiz, Spain) to be used for this study.

Commercial crude borage oil (from Apsara Vital, Barcelona, Spain) was also used as feed material to be fractionated by means of a countercurrent column.

The carbon dioxide (99%) used was provided by Carburos Metalicos (Barcelona, Spain). The methyl ester standard (99%) which include methyl palmitate, methyl stearate, methyl oleate, methyl linoleate, methyl γ-linolenic and methyl heptadecanoate as internal standard; were purchased from Sigma-Aldrich (Barcelona, Spain). The 2,2-diphenyl-1-picrylhydrazyl (DPPH) also was provided by Sigma-Aldrich (Barcelona, Spain). The rest of the reagents (ethanol, toluene, potassium hydroxide 0.1 mol/L, phenolphthalein solution, ethyl acetate and methanol) were supplied by Panreac.

### 2.2. Extraction and Fractionation at High Pressure

**Figure 1:**
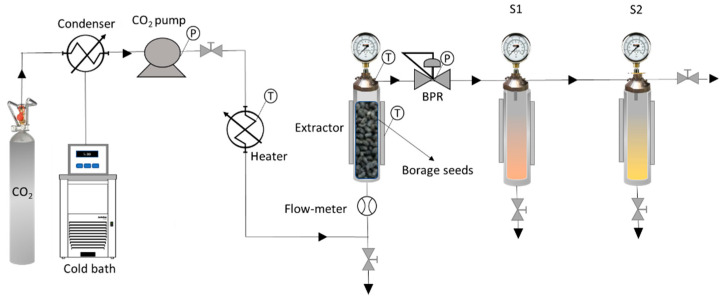
Simplified diagram of the supercritical extraction and fractionation equipment (BPR: automated back pressure regulator, S1 and S2: cyclonic separators, T: temperature, P: pressure).

The extractions experiments were carried out by means of a Thar Technology tool (Pittsburgh, PA, USA, model SF2000), fitted with an extractor (2 L capacity), a high-pressure pump for CO_2_ (P200 model) and two cyclone separators (500 mL each) laid out in series. Figure 1 is a diagram of the equipment used for this research. A more detailed description of the equipment and its operating methodology can be found in a previous publication [28].

The extractor was loaded with 550 g of raw material. The extraction conditions in the extractor (400 bar/55 °C) were selected on the basis of previous works [16,28,29]. The use of high pressures (400–500 bar) and a relative high temperature (50–55 °C) led to high extraction yields. The saturated SC-CO_2_-extract mixture at the exit point of the extraction vessel first reached separator 1, which was maintained at a lower pressure than the extraction vessel. As the operating pressure was changed, the solubility of some of the ingredients in the mixture of SC-CO_2_ and extract diminished, which resulted in their precipitation into the cyclone separator 1 (S1). The mixture of SC-CO_2_ and the remaining part of the extract then reached the cyclone separator 2 (S2), which was maintained at atmospheric conditions, for the recovery of the remaining compounds. Four sets of extract fractionations obtained under different conditions were analysed (Table 1):

After the extraction time (4 h), the separators were depressurized, and the fractions were collected in flasks and stored at 4 °C in the absence of light for further analysis. The experiments on each sample were carried out in duplicate in order to determine the variability of the measurements. The total yields obtained were calculated as the total extracted mass divided by the dry raw material mass.

### 2.3. Supercritical Fluid Fractionation Column

**Figure 2:**
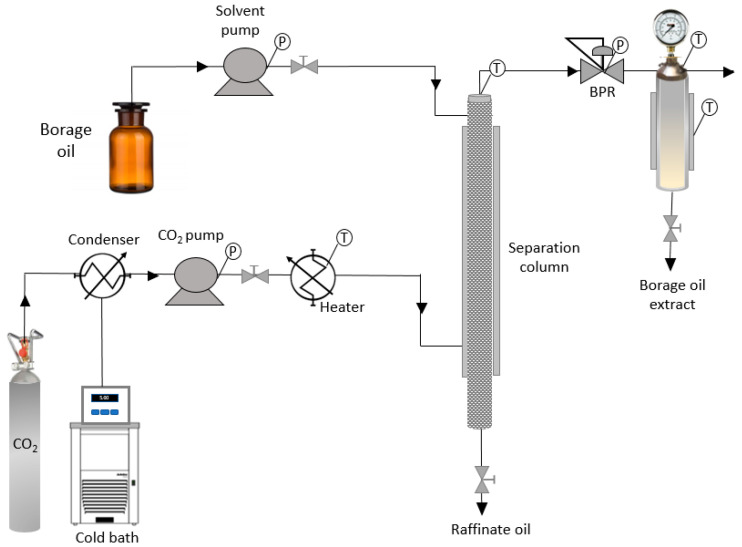
Simplified diagram of the countercurrent supercritical fluid fractionation column (BPR: automated back pressure regulator, S1 and S2: cyclonic separators, T: temperature, P: pressure).

The fractionation column experiments were carried out by means of a Thar Technology tool (Pittsburgh, PA, USA, countercurrent packed column), fitted with a stainless-steel column (1.765 L capacity), two high pressure pumps: one for CO_2_ (P200 model) and the other one for the oil (P50 model), and a cyclone separator (500 mL). Figure 2 is a simplified diagram of the equipment used for the experiments in this research.

The column was 2.8 m long and 2 cm diameter, making up 1.765 L of total volume. The column was packed with stainless steel packing material (Propak perforated flakes). It was also fitted with three side inlets: one at the bottom, to add the solvent (SC-CO_2_), one in the middle and one at the top, to inject the feed (borage oil). The column was also fitted with two outlets, one at the top (extract exit) and one at the bottom (to remove the raffinate). The column temperature was also controlled by means of four sensors in four different areas along its body and a manometer was used to monitor the system pressure.

The lower section of the column, 30 cm below the CO_2_ inlet, was used as raffinate reservoir. The extract was collected into the cyclone separator. The temperature and pressure of the separator was monitored by means of a manometer and a temperature sensor connected to the control device. The pressure conditions were controlled by means of a back-pressure regulator at the top end of the column.

The equipment was run in automatic mode and controlled by computer using the software ICM (Thar Tech. Pittsburgh, PA, USA). This application allows you to carry out countercurrent extractions at constant pressure and temperature.

The fractionation experiments were carried out at 100, 200, 300 and 400 bar of pressure and temperatures of 35, 55 and 75 °C. The carbon dioxide and the oil flow rates were 20 g/min and 3 g/min, respectively. After completing each experimental run the column was depressurized and the residual oil was drained. The operating time for each assay was 4 h.

The extract and the raffinate were collected in flasks and stored at 4 °C away from the light for further analysis. The experiments were carried out in duplicate for each set of conditions to determine the variability of the measurements.

### 2.4. Acidity Index

The acidity index of the fractions was determined according to UNE-55011. The acidity index is the mass, expressed in mg, of potassium hydroxide that is necessary to neutralize the free fatty acids (FFA) contained in 1 g of sample. It is generally represented by the percentage of oleic acid, which is the most abundant FFA [40].

### 2.5. Determining by DPPH the Antiradical Activities of the Potent Antioxidants

The method used to measure the antioxidant activity of the fractions obtained was based on the use of DPPH as a free radical. The technique proposed by Brand-Williams et al. [41], and modified by Scherer et al. [42], is based on the absorbance of the free radical 2,2-diphenyl-1-picrylhydrazyl (DPPH) measured at 515 nm. The reduction of this radical with the presence of antioxidants leads to eliminating the absorption at this wavelength. Thus, the degree of reduction of the absorbance allows to determine the ability of the compound to scavenge free radicals.

The 3.9 mL of 6 × 10^−5^ mol/L ethyl acetate DPPH solution were added to 0.1 mL of an oil solution in ethyl acetate at different concentrations (50–350 mg/mL). The blank sample consisted of 0.1 mL of ethyl acetate added to 3.9 mL of DPPH solution.

The EC_50_ (efficient concentration providing 50% inhibition) was graphically estimated based on a non-linear curve representing the %DPPH remaining at the steady state vs. the oil concentration. The experiments were carried out in duplicate to determine the variability of the measurements.

### 2.6. Fatty Acids Content

The fatty acids content was determined by gas chromatography (GC) by means of an Agilent Technologies chromatograph 6890 N model fitted with a TR-CN100 capillary column (60 m length × 0.25 mm internal diameter × 0.20 μm thickness) and a flame ionization detector. The injector and detector temperatures were 280 °C and 260 °C, respectively. The oven temperature was 185 °C. The carrier gas used was hydrogen at the rate of 38.02 cm/s, and air and hydrogen were used as auxiliary gases [43].

The fatty acids were identified by comparing the retention times of the borage methyl esters against those of known mixtures of standard fatty acids (Sigma-Aldrich, Barcelona, Spain) after processing them with the same column under the same conditions.

A preliminary step was required prior to injecting the oil into the GC for the individual determination of its fatty acids content. The fractions obtained were converted into their corresponding methyl ester (FAME) [44].

### 2.7. Cell Cultures

The human leukaemia cell line HL60 (promyelocitic cells) was supplied by Dr. Angeles Alonso-Moraga (Genetic Department, University of Cordoba, Spain). HL60 cells were grown in a RPMI-1640 suspension medium (Invitrogen, Waltham, MA, USA). This medium was supplemented with antibiotics 100× (Sigma, Darmstadt, Germany), L-glutamine 200 mM (Sigma, Darmstadt, Germany) and 10% heat-inactivated foetal bovine serum (Linus, Cultek, Madrid, Spain). The experiments were run in a 5% CO_2_ humidified atmosphere at 37 °C using a CO_2_ Incubator (Shel Lab, Sheldon, OR, USA). The HL60 cells were subcultured every 2–3 days to maintain their logarithmic growth and were allowed to grow for 48 h before use. The cultures were plated at a density of 12.5 × 10^4^ cells mL^−1^ in 40 mL culture bottles (25 cm^2^).

### 2.8. Cytotoxicity Assays

The cytotoxic activity of the different oil samples and extracts was measured as the growth inhibition or decreased viability of the human promyelocytic leukaemia cell line HL60. In order to measure the cytotoxic effect of the tested samples, the protocol proposed by Zhu and Loft [45] with some modifications was used. Briefly, cells were seeded at 10^5^ cells/mL on 12- or 96-well plates and incubated in the different concentrations of oil fractions for 72 h prior to being subjected to the trypan blue exclusion assay (Sigma, Darmstadt, Germany). The reactivity of trypan blue, a vital dye, is based on the fact that the chromopore is negatively charged and does not interact with the cells unless their membranes are damaged. Therefore, while viable cells remained unstained, non-viable cells get stained with a purple-violet colour. Trypan blue was added to cell cultures at a 1:1 ratio, and 20 μL of the cell suspension was loaded into a Neubauer chamber. The cells were counted under an inverted microscope (Motic, AE30/31, MoticEurope S.L.U., Barcelona, Spain) at ×100 magnification.

After each culture incubation period, a growth curve was established and the IC_50_ values (concentration of tested compound causing 50% inhibition of cell growth) were estimated. The lower IC_50_ the higher the efficacy of the substance against tumour growth. The curves were expressed as the cell survival percentage with respect to that of the control samples after 72 h of growth. The viability curves of the leukaemia cells were plotted as their mean viability ± standard error of at least three independent replicas of each treatment and concentration.

## 3. Results

Two fractionation techniques were applied to borage oil together with SC-CO_2_: precipitation into two cyclone separators and countercurrent extraction column. The acidity index, antioxidant capacity and cytotoxic effect against promyelocytic HL60 leukaemia cells of the different fractions obtained were also determined.

Most cancer cells remain in a highly proliferative state that outgrows their normal cellular counterparts. An alternative approach to the destruction of cancer cells by conventional antineoplasic agents consist in the implementation of biological sub-stances that can induce cytotoxicity or, alternatively, a terminal differentiation and apoptosis [46,47,48]. Therefore, the induction of cell death in proliferative tumour cells may represent an efficient approach to cancer chemotherapy [49]. Human promyelocytic leukaemia cell line HL60 has been selected as the model for a wide range of substances with the potential to be used as antineoplasic agents and has proved to be a valid testing system to monitor massive preclinical screening experiments [50,51,52,53,54].

### 3.1. Fractionation of the Extracts into Cyclone Separators

The supercritical fluid fractionation of borage oil seeds was performed by means of two cyclone separators, followed by a step to purify the oil. The whole procedure involved the stepwise precipitation of the fractions obtained from the extraction. This was achieved by modifying the density of the CO_2_ through changes in the pressure and temperature in the separators. The density data can be seen in Table 2. Through this method, the compounds that were least soluble in the supercritical solvent precipitated into the first separator while the more soluble materials reached the second separator [28,29,30].

Table 2 shows the extraction yields collected into each separator according to the varying S1 temperature and pressure conditions. In the case of the run conducted at 70 bar and 40 °C practically all the extract was collected into S1. Therefore, no fractionation was obtained from borage oil seeds under these conditions. The yield extraction collected into S1 increased to 34% at 200 bar/45 °C and up to 82% at 90 bar/40 °C. These results indicate that the pressure and temperature conditions at S1 were suitable to obtain different extraction rates. Additionally, the results would be later compared against the seed oil obtained by applying a conventional extraction technique where hexane would be used as the solvent in a Soxhlet equipment. The extraction yield was similar (22.0 ± 4 wt.%) to that obtained by supercritical fractionation. In order to evaluate the fractionation process, the fractions were characterized by measuring their acidity index, antioxidant activity, fatty acids composition and cytotoxicity.

Depending on biological, meteorological, and agricultural factors or on processing conditions, the composition of fatty acid content (FFA) can vary significantly. These substances are susceptible to oxidation. A phenomenon that results in an undesirable rancid flavour of the oil. Consequently, large amounts of free fatty acids in the oil should be definitely avoided, since the lower the free fatty acids content the higher the oil’s commercial value. The deacidification of crude borage oil is important not only with regard to consumer’s acceptance but also because it has a definite impact on the economic side of its production. The removal of FFA from crude oil is, therefore, a crucial aspect of oil production since it largely determines the quality of the final product [55,56].

Table 2 presents the acidity index of the oil collected into each separator with respect to the different fractionation conditions. It can be seen from these data that there is a wide variation between the acidity indexes of the oil in each separator (2.7–10.5%). The acidity index measured in S1 (2.93% mean value) does not significantly change with the different fractionation conditions (~4%) but all the values obtained are significantly lower than the acidity index of the raw material 5.4% (see the result obtained at 70 bar and 40 °C when no fractionation is achieved). These results indicate that the oil fractionated by this method at pressures over 70 bar has a lower concentration of free fatty acids. On the other hand, the oil obtained in S2 (7% mean value) when fractionated at 90 bar and 40 °C present the highest concentration of free fatty acids, which increases the acidity index of these fractions. On average, and regardless of the fractionation conditions, the S1 separator reduced from 7% to 2.93% the acidity of the resulting extract (approx. 2.4 times), which represents a considerable added value to the SC oil.

The tocopherols and phenolic compounds are known to be the most important natural antioxidants in oils. According to scientific evidence the tocopherol and phenolic contents vary depending on the oil extraction process applied in each case [57,58,59]. It is therefore important to evaluate the antioxidant activity of the different fractions obtained. Table 2 includes the EC_50_ value of the different fractions that were collected into the cyclone separators. Lower EC_50_ values imply higher antioxidant activity levels. The slight differences between the results registered for each separator suggests that the EC_50_ of the oil in S1 (135% mean value) is lower than that corresponding to the oil in S2 (174% mean value) and would indicate around 20% less antioxidant activity.

The fatty acids composition of the borage oil separated fractions was studied by gas chromatography. Table 3 presents these results. The S1 and S2 fractions obtained under different conditions exhibited the same proportions of fatty acids, as no differential pattern was observed. To analyse the significance of each fatty acids into the different separators a statistical analysis (ANOVA) of the results was carried out. The *p*-value are included in Table 3; in all cases *p*-value was greater than 0.05. Therefore, the cascade fractionation method is not selective regarding fatty acids.

**Figure 3:**
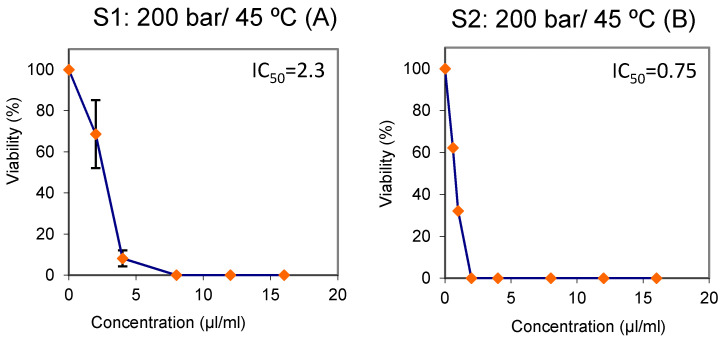

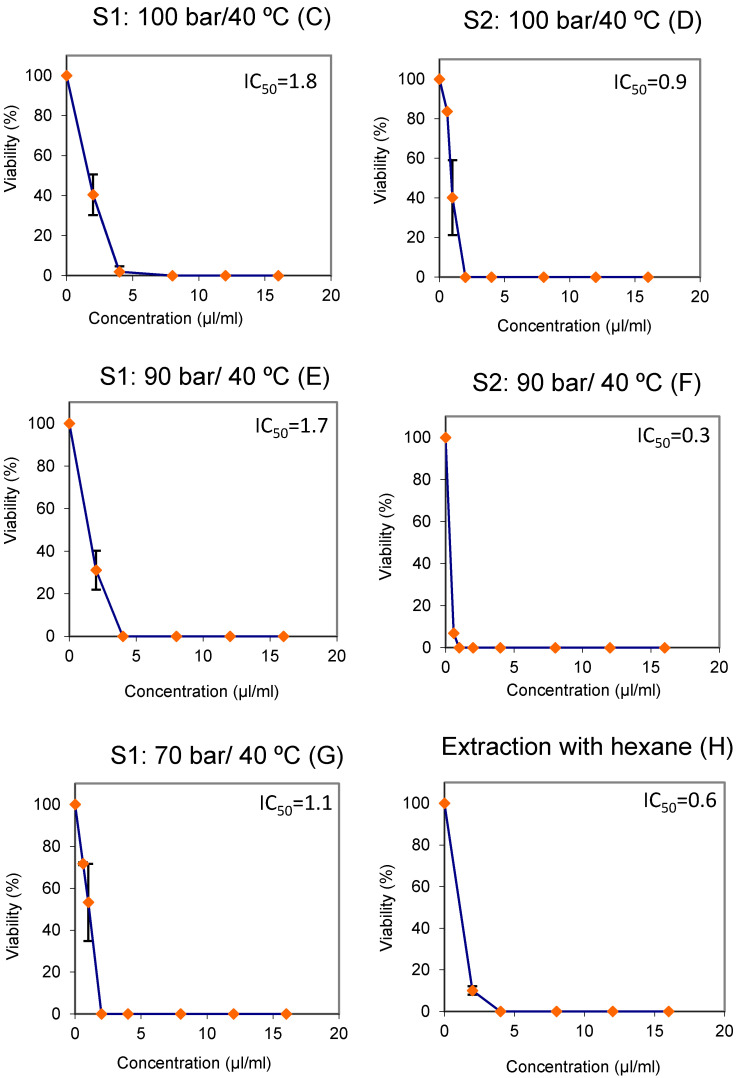
Viability of HL60 cells treated for 72 h either with the extracts collected into each one of the two separators; S1 (**A**,**C**,**E**,**G**) and S2 (**B**,**D**,**F**) or with hexane (**H**). The curves represent the percentages with respect to the results obtained from three independent experiments on the control substance (mean ± SD).

Finally, Figure 3 includes the cell growth percentage of the fractions with respect to the control samples after 72 h. The graphs represent the IC_50_ values obtained from the cytotoxicity assays on the oil from each one of the two separators. The results indicate that the oil from both separators exhibit a substantial inhibitory activity against tumour cells’ growth that largely depends on the dose applied, with IC_50_ values ranging from 0.3 up to 2.3 μL/mL. The lowest IC_50_ values (greater cytotoxicity) corresponded to the oil from separator S2, regardless of the fractionating conditions. In fact, the concentration level required to reach IC_50_ by the S1 extract was higher than that required by the S2 extract, regardless of the specific fractionating conditions. On the other hand, the tumouricide activity by the extracts collected into S1 under different pressure/temperature conditions were higher when pressure/temperature conditions were lower (70 bar/40° Figure 3G) than under the highest pressure/temperature conditions tested (Figure 3A,C,E). The same can be said for the extracts collected into S2 (Figure 3B,D,F). Thus, the extract from S2 obtained at 90 bar/40 °C showed the highest anti-proliferative activity in low doses, with an IC_50_ of only 0.3 μL/mL, and 100% inhibition when a concentration of just 1 μL/mL was applied (Figure 3F). The oil obtained under these conditions exhibited a high concentration of free fatty acids (high acidity index) and seem to be the most rapid and effective of all the extracts tested against the exponential growth of HL60 promyelocytic cells. The extract obtained by Soxhlet extraction also exhibit a substantial inhibitory activity against tumour cells’ growth (Figure 3H).

### 3.2. Countercurrent Fractionation of the Oil

Table 4 includes the values of the extract and raffinate flowrates under all the crude oil extraction conditions tested. Depending on the solubility of the oil components in SC-CO_2_, the extract or raffinate flowrate would be higher or lower, but their sum total should always match the total feed flowrate. The oil components which were not significantly solubilised in the SC-CO_2_ settled into the raffinate reservoir at the bottom of the column. An increment of the extract flowrate took place as pressure was increased and temperature decreased.

**Figure 4:**
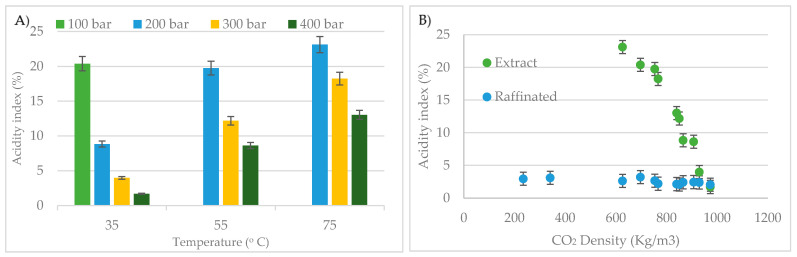
Effect of temperature and pressure (**A**) as well as density (**B**) on the acidity index of the crude borage oil fractions (mean ± SD).

Figure 4A shows the extract acidity index values according to the different pressure and temperature conditions studied. The highest acidity index (20.4%) corresponds to the fractions obtained at 35 °C and under 100 bar. This index decreased as pressure was increased, with values as low as 1.61% when operating under 400 bar. A similar trend was observed when the operating temperature was 55 °C or 75 °C. Therefore, it can be concluded that under isothermal conditions pressure increments result in lower extract acidity indexes. On the other hand, under isobaric conditions, temperature increments give rise to higher extract acid indexes. Both trends are related to the density of the supercritical CO_2_, thus in Figure 4B the extract and the raffinate’s acidity indexes have been plotted as a function of the CO_2_ density. It can be seen that the acidity index of the raffinate is independent from the operating conditions since it maintains a constant value of 2.6 ± 0.5%. However, a marked decrease in the acidity of the extract is detected with increasing solvent density. The greatest differences and, therefore, the most selective fractionations, were registered at intermediate density values (600–800 Kg/m^3^).

Table 5 shows the antioxidant activity of the raffinate oil expressed as EC_50_ values. When comparing these results against the EC_50_ value of the initial crude oil, which was 320 mg/mg DPPH, a fall is observed and, therefore, an increment in the antioxidant capacity of this fraction is confirmed. The average values of the raffinates obtained at 35 °C, 55 °C and 75 °C were 254.3, 266.5 and 255.25 mg/mg DPPH, respectively.

The lipids in crude borage oil, which are mainly triglycerides, may deteriorate as a consequence of the chemical reactions that take place at the high temperatures required for refining processes. In order to determine the degree of deterioration, the triglyceride profile of the raffinates should be determined and compare against that of the crude oil. Table 6 shows a comparison between the triglyceride profiles of the raffinate, the extract stream and the feed oil. ANOVA analysis shows that practically all the *p*-values of the F-test are greater than 0.05 (Table 6). For these reasons not a statically significant difference at the 5% significance level. Only the palmitic acid data show a significant difference in the extract.

We can see that they present similar profiles, which seems to indicate that the oil quality has not been seriously affected by its supercritical processing. Linoleic, oleic and γ-linolenic acids represent most of the fatty acid content in the raffinate.

**Figure 5:**
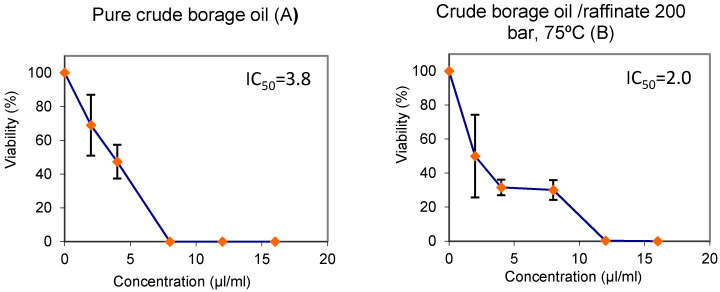
Viability of HL60 cells treated for 72 h either with pure crude borage oil (**A**) or with raffinate borage oil (**B**). The curves represent the percentages with respect to the results obtained from three independent experiments on the control substance (mean ± SD).

Finally, Figure 5 below shows the results from the cytotoxicity assays carried out on the samples of unfractionated and fractionated oil at 200 bar and 75 °C. Again, the significant inhibition values exhibited by the two samples should be noted. Different trends and inhibitory concentration values (IC_50_) were obtained from the two borage oils tested, crude and raffinated under 200 bar at 75 °C (Figure 5A,B). The lethal dose (IC_50_) was reached at a concentration of 3.8 µL/mL in the case of the crude borage oil (Figure 5A), whereas IC_50_ was reached at a concentration of 2 µL/mL by the raffinate obtained from this oil (200 bar/75 °C). The HL60 growth curves present different shapes (Figure 5A,B), since in the case of the crude borage oil 100% inhibition was registered at just 8μL/mL concentration, while the raffinate required a concentration of 12 μL/mL) to reach the same inhibitory level.

## 4. Discussion

Lipid extracts generally obtained by SC-CO_2_ contain many lipophilic compounds, and the complexity of such systems represent a challenge for fractionation processes. In the present work, borage oil is fractionated through two different techniques, cascade and countercurrent, both of them applied after the oil extraction by means of SC-CO_2_. The antioxidant and cytotoxic properties of the fractions obtained have been determined. Cascade fractionation was performed directly from borage seeds, and countercurrent packed column was applied to borage crude oil.

In relation to the cascade fractionation process, it was observed that a decrease of the CO_2_ density from 865.66 to 198.32 kg/m^3^ caused an increment in the percentage of the extract obtained in S1 (Table 2) which became practically a single fraction under 70 bar/40 °C. Therefore, the operating conditions established for S1 allowed to obtain notably different separation percentages of the fractions. This large increment does not take place with every type of extract. For example, Tamkute et al. investigated the possibility of fractionating cranberry pomace after its extraction with SC-CO_2_ with and without 5% ethanol under previously optimized conditions consisting in 424 bar pressure and 53 °C temperature [60]. The extract was separated into a heavier (S1) and a lighter (S2) fraction by reducing the temperature in S1 from 0 to −30 °C while pressure remained constant at 70 bar. When extraction was performed without ethanol, 61–74% of the total extract was collected into S1 and the remaining 26–39% into S2. When the supercritical extraction was performed with CO_2_ + 5% ethanol, the cooling effect from the separator was more temperature dependent. They concluded that the fractionation phenomena are difficult to explain because of the complexity of the system, which involves a large number of compounds from a diversity of chemical classes and with different properties [60].

The oil extracted directly from borage seeds with hexane by means of a standard Soxhlet equipment exhibited an acidity index of 3.2 ± 0.2, which is lower than that exhibited by the crude extract (5.4 ± 0.1%) (Table 2). The oils extracted using SC-CO_2_ may have higher free fatty acid and peroxide levels [61,62] than those obtained through conventional extractive methods. In our study, the results from the characterisation of the fractions that had been collected into each one of the separators make us think that most of the undesirable free fatty acids are collected into the second separator. This should be considered as one of the advantages of a cascade fractionation method.

The antioxidant activity of oils may be attributed to their chemical constituents such as polyunsaturated fatty acids, sterols, tocopherols, and polyphenols [61]. Oils extracted using SC-CO_2_ tend to have low levels of antioxidants due to the low solubility of their constituents into in pure CO_2_. In general, according to our results (Table 2) the tested oils present better antioxidant capacity than other oils mentioned in the literature, such as walnut oil (1514 mg/mg DPPH), almond oil (712 mg/mg DPPH), hazelnut oil (478 mg/mg DPPH) peanut oil (1395 mg/mg DPPH) or pistachio oil (377 mg/mg DPPH) [63,64], which suggests that borage oils might play a more prominent role as an antioxidant and health-promoting substance.

The lipids that contain long-chain polyunsaturated fatty acids are highly appreciated these days as we grow aware of the beneficial effects of ω-3 and ω-6 fatty acids for the treatment of certain ailments, and as a health promoter in general. Cascade fractionation does not result into significantly different fatty acid compositions and, therefore, the quality of the oils remains the same for both fractions. No noticeable differences can be observed when these oils are compared against those obtained by Soxhlet extraction. These results are in agreement with previously published works on this matter [63,64].

Finally, the results from the cytotoxicity assays on the cascade fractionates indicate that both products have significant inhibitory activity against tumour cell growth. However, the oil collected into the second separator present slightly lower IC_50_ values. This suggests that the substances that are responsible for the antitumor properties of the oil are highly soluble in carbon dioxide and, therefore, reach the second cyclone separator while solved in the SC-CO_2_ flow. As above mentioned, CO_2_ high density values are associated to a higher solvent power of the SC-CO_2_. Other studies on the cytotoxic activity of commercial borage oils reported similar IC_50_ value (1 μL/mL) [15]. The higher acidity indexes determined for the S2 fractions are qualitatively and directly correlated with their IC_50_ values and inversely correlated with their antioxidant activity. HL60 cells have generally been used for anticancer screening purposes in food research studies. Several substances, either simple molecules or more complex mixtures, have been reported to exhibit a high cytotoxic activity against this cell line that share an acidic profile. Gamma-linolenic acid, lemon juice or cola beverage extracts [15,65,66] belong to this group of compounds. Based on these findings, we have hypothesised that the higher cytotoxic activity exhibited by the S2 fractions could be due to the notably higher acid residues that can be found in them.

Regarding the analysis of the fractions obtained through the countercurrent column method, the extract acidity index increments that take place as the density of the supercritical carbon dioxide is increased, clearly correlate with the lower acidity indexes of the raffinate. This can be explained by the higher density of the SC-CO_2_ under higher pressures and at lower temperatures, with more pronounced differences at the SC-CO_2_ lowest density levels. This high value in density is related to high values in the solvent power of SC-CO_2_. Similar results were reported by previous works. Thus, Simões et al. evaluated the quality of an olive oil refined by supercritical fluid fractionation at 180 bar/40 °C and 260 bar/80 °C [67]. The data from their analyses showed that supercritical carbon dioxide does not alter the nutritional quality of the oil. However, the extensive deacidification that took place would result in a poor acid content of the raffinates. Similar results were reported by Vázquez et al., who used countercurrent supercritical carbon dioxide extraction at 40 °C and 180, 234 and 250 bar to remove free fatty acids from cold-pressed olive oil [68].

On the other hand, the DPPH data registered agree with those determined for the cascade fractionation method. Thus, the acidity indexes of the raffinate oil stream surpassed those corresponding to the crude oil. Considering the yields obtained and their corresponding DPPH values, 200 bar and 75 °C were the selected conditions for the cytotoxicity assays. Again, it was observed that oil fractionation significantly improved tumour cell growth inhibitory activity when compared to that of the crude oil extract.

## 5. Conclusions

Borage oil has already been confirmed as a product with excellent health-promoting properties. Its high content of polyunsaturated fatty acids makes of this oil an excellent dietary product. Conventional refining and other processes tend to degrade oil in a manner that affects its beneficial properties. It is, therefore, necessary to explore alternative methods that are more environmentally friendly and that do not alter the properties to be expected from the final product. In the present work, two fractionation methods after applying supercritical carbon dioxide for the oil extraction have been analysed: cascade cyclone separators and countercurrent column. The results obtained by either method have been quite satisfactory, since the untreated oil fatty acid profile remained unaltered, and the resulting products presented lower acidity and exhibit improved antioxidant and cytotoxic properties when compared to those of the crude oil. Fractionated oils can be used directly as a dietary supplement and are an excellent source of polyunsaturated fatty acids that could complement other food products.

## Figures and Tables

**Table 1:** Fractionation conditions of the two cyclone separators.

Test	Separator 1 (S1)	Separator 2 (S2)
1	200 bar/45 °C	atmospheric conditions
2	100 bar/40 °C	atmospheric conditions
3	90 bar/40 °C	atmospheric conditions
4	70 bar/40 °C	atmospheric conditions

**Table 2:** Properties of the extracts obtained by cascade fractionation (S1: separator 1 and S2: separator 2). Data are represented as the mean with the 95% confidence intervals.

Fractionation Conditions	CO_2_ Density (kg/m^3^) in S1	Separation Percentage (%)		Acidity Index (%)		EC_50_ Values (mg/mg DPPH)	
		S1	S2	S1	S2	S1	S2
200 bar/45 °C	865.66	34 ± 4	66 ± 3	2.7 ± 0.6	3.5 ± 1.2	150 ± 7	178 ± 7
100 bar/40 °C	622.64	55 ± 3	45 ± 2	3.1 ± 0.9	7.0 ± 1.2	132 ± 8	182 ± 4
90 bar/40 °C	484.09	82 ± 6	18 ± 4	3.0 ± 0.8	10.5 ± 1.9	138 ± 6	162 ± 9
70 bar/40 °C	198.32	99± 1	1± 1	5.4 ± 0.6	n.d.	118 ± 7	n.d.

n.d. property not detected.

**Table 3:** Composition of the fatty acids (%) in the different fractions obtained into the different separators (S1: separator 1 and S2: separator 2). Data are represented as the mean with 95% confidence intervals.

Fractionating Conditions		Palmitic	Estearic	Oleic	Linoleic	γ-Linolenic	Eicosenoic	Erucic
		C16:0	C18:0	C18:1	C18:2	C18:3 (n-6)	C20:1	C22:1
200 bar/45 °C	S1	11.30 ± 1.50	4.07 ± 0.64	16.51 ± 1.91	37.21 ± 2.51	24.01 ± 2.25	4.11 ± 1.33	2.80 ± 1.91
	S2	11.76 ± 1.03	3.86 ± 0.64	16.78 ± 1.27	37.22 ± 1.99	24.07 ± 2.94	3.79 ± 1.09	2.53 ± 1.89
100 bar/40 °C	S1	11.50 ± 1.64	3.89 ± 0.99	16.53 ± 1.27	37.29 ± 2.34	24.27 ± 3.03	3.94 ± 1.65	2.58 ± 1.67
	S2	12.89 ± 1.82	4.21 ± 0.81	17.29 ± 1.49	36.53 ± 2.12	23.11 ± 2.78	3.60 ± 1.69	2.36 ± 1.50
90 bar/40 °C	S1	11.66 ± 1.95	3.84 ± 0.94	16.54 ± 1.31	37.31 ± 2.63	24.18 ± 2.69	3.81 ± 2.36	2.69 ± 1.56
	S2	13.35 ± 1.91	4.40 ± 0.64	17.68 ± 1.23	35.78 ± 2.54	22.77 ± 3.03	3.53 ± 02.29	2.51 ± 1.49
70 bar/40 °C	S1	11.84 ± 1.92	3.93 ± 1.03	16.54 ± 1.44	37.13 ± 2.09	24.16 ± 2.38	3.80 ± 02.67	2.62 ± 1.82
Extraction with hexane		10.15 ± 1.88	4.12 ± 0.64	17.18 ± 1.23	38.01 ± 2.45	24.11 ± 2.94	4.18 ± 2.40	2.63 ± 1.49
ANOVA/*p*-value		0.0678	0.3021	0.289	0.129	0.5673	0.3478	0.5871

**Table 4:** Extract and raffinate flowrates (g/min) registered when fractionating crude borage oil by means of a countercurrent column technique.

Pressure	Extracts 35 °C	Raffinate 35 °C	Extracts 55 °C	Raffinate 55 °C	Extracts 75 °C	Raffinate 75 °C
100 bar	0.005	4.950	0	5.297	0	4.877
200 bar	0.071	5.536	0.037	5.556	0.004	5.643
300 bar	0.550	5.750	0.133	5.000	0.100	5.769
400 bar	5.250	0	0.360	4.950	0.278	4.444

**Table 5:** Antioxidant capacity of the raffinate oil determined by DPPH (EC_50_ expressed as mg/mg DPPH with the 95% confidence intervals).

Pressure	Raffinate 35 °C	Raffinate 55 °C	Raffinate 75 °C
100 bar	278 ± 7	240 ± 5	270 ± 8
200 bar	237 ± 5	259 ± 7	251 ± 5
300 bar	248 ± 5	283 ± 4	247 ± 4
400 bar	-	284 ± 7	253 ± 6

**Table 6:** Fatty acid compositions (%) of the different fractions obtained through countercurrent column under 200 bar at 75 °C. Data are represented as the mean with 95% confidence intervals (* significat differences).

Fractionation Conditions	Palmitic	Estearic	Oleic	Linoleic	γ-Linolenic	Eicosenoic	Erucic
	C16:0	C18:0	C18:1	C18:2	C18:3 (n-6)	C20:1	C22:1
Crude borage oil	10.69 ± 1.97	4.61 ± 0.97	18.63 ± 2.91	37.45 ± 2.76	21.22 ± 3.01	4.34 ± 1.39	3.08 ± 1.17
Raffinate	10.60 ± 2.25	4.57 ± 0.88	18.66 ± 2.76	37.52 ± 3.05	21.34 ± 2.98	4.40 ± 1.48	2.94 ± 1.18
Extract	13.18 * ± 1.09	4.36 ± 0.91	20.02 ± 1.03	37.27 ± 2.93	19.87 ± 2.75	3.34 ± 1.26	1.96 ± 1.06
ANOVA/*p* value	0.036	0.5482	0.2138	0.9923	0.6237	0.1523	0.5488

## Data Availability

All the data presented in this study are available in this article.
